# Supplementary material for: Structure-guided identification of mitogen-activated protein kinase-1 inhibitors towards anticancer therapeutics
Source: PLoS One. 2025 Jan 24;20(1):e0311954. doi: 10.1371/journal.pone.0311954 (PMC11760640; doi:10.1371/journal.pone.0311954)
Supplement: S1 Table — (DOCX) [file pone.0311954.s001.docx]

**Structure-guided identification of mitogen-activated protein kinase-1 inhibitors towards anticancer therapeutics**

Md Nayab Sulaimani^1^, Shazia Ahmed^2^, Farah Anjum^3^, Taj Mohammad^1^, Anas Shamsi^4,*^, Ravins Dohare^1*^, and Md. Imtaiyaz Hassan^1,*^

*^1^Centre for Interdisciplinary Research in Basic Sciences, Jamia Millia Islamia, New Delhi 110025, India.*

*^2^Department of Computer Science, Jamia Millia Islamia, New Delhi 110025, India.*

*^3^Department of Clinical Laboratory Sciences, College of Applied Medical Sciences, Taif University, PO Box 11099, 21944, Taif, Saudi Arabia.*

*^4^Center of Medical and Bio-Allied Health Sciences Research (CMBHSR), Ajman University, Ajman, United Arab Emirates.*

*Running Head: MAPK1 inhibitors*

****Correspondence should be addressed to:***

**Dr. Anas Shamsi**

E-mail: anas.shamsi18@gmail.com

**Prof. Ravins Dohare**

Centre for Interdisciplinary Research in Basic Sciences

Jamia Millia Islamia, New Delhi 110025, India.

E-mail: [ravins@jmi.ac.in](mailto:ravins@jmi.ac.in)

**Dr. Md. Imtaiyaz Hassan, Ph.D., FRSB, FRSC.**

Centre for Interdisciplinary Research in Basic Sciences

Jamia Millia Islamia, New Delhi-110025, India

E-mail: [mihassan@jmi.ac.in](mailto:mihassan@jmi.ac.in)

**Table S1:** List of filtered docked output^*^.

| Name of the ligand | Binding Free Energy (kcal/mol) | p*K*i | Ligand Efficiency (kcal/mol/non-H atom) | Torsional Energy |
| --- | --- | --- | --- | --- |
| ZINC03845566 | -12 | 8.8 | 0.3158 | 0 |
| ZINC02161110 | -11.6 | 8.51 | 0.3053 | 0.9339 |
| ZINC02133365 | -11.3 | 8.29 | 0.2974 | 1.2452 |
| ZINC02161108 | -11.2 | 8.21 | 0.2947 | 0.9339 |
| ZINC03844856 | -11.1 | 8.14 | 0.3083 | 0.9339 |
| ZINC04235984 | -11.1 | 8.14 | 0.2921 | 1.2452 |
| ZINC02092851 | -11 | 8.07 | 0.3056 | 1.2452 |
| ZINC02130275 | -11 | 8.07 | 0.2895 | 1.2452 |
| ZINC04235928 | -11 | 8.07 | 0.2973 | 1.2452 |
| ZINC04235931 | -11 | 8.07 | 0.3143 | 1.2452 |
| ZINC04235932 | -11 | 8.07 | 0.2973 | 1.5565 |
| ZINC04236439 | -11 | 8.07 | 0.3056 | 1.5565 |
| ZINC02161106 | -10.9 | 7.99 | 0.2868 | 0.9339 |
| ZINC02095133 | -10.9 | 7.99 | 0.3028 | 1.2452 |
| ZINC05415116 | -10.9 | 7.99 | 0.3114 | 1.2452 |
| ZINC02160906 | -10.9 | 7.99 | 0.3206 | 1.5565 |
| ZINC03839304 | -10.9 | 7.99 | 0.2946 | 1.5565 |
| ZINC04260971 | -10.9 | 7.99 | 0.2868 | 1.8678 |
| ZINC03999625 | -10.8 | 7.92 | 0.3484 | 0.6226 |
| ZINC04083886 | -10.8 | 7.92 | 0.3176 | 0.9339 |
| ZINC02119958 | -10.8 | 7.92 | 0.3375 | 1.2452 |
| ZINC02133362 | -10.8 | 7.92 | 0.2842 | 1.2452 |
| ZINC02133367 | -10.8 | 7.92 | 0.2842 | 1.2452 |
| ZINC03839280 | -10.8 | 7.92 | 0.2919 | 1.5565 |
| ZINC03839406 | -10.8 | 7.92 | 0.2919 | 1.5565 |
| ZINC04270628 | -10.8 | 7.92 | 0.2919 | 1.5565 |
| ZINC01960942 | -10.8 | 7.92 | 0.3 | 2.1791 |
| ZINC02092966 | -10.8 | 7.92 | 0.2919 | 2.1791 |
| ZINC04083885 | -10.7 | 7.85 | 0.3147 | 0.9339 |
| ZINC03839376 | -10.7 | 7.85 | 0.2972 | 1.2452 |
| ZINC03839453 | -10.7 | 7.85 | 0.2816 | 1.2452 |
| ZINC02128796 | -10.7 | 7.85 | 0.3057 | 1.8678 |
| ZINC04278032 | -10.7 | 7.85 | 0.3057 | 1.8678 |
| ZINC03839446 | -10.6 | 7.77 | 0.2865 | 0.9339 |
| ZINC03848911 | -10.6 | 7.77 | 0.3655 | 0.9339 |
| ZINC02120692 | -10.6 | 7.77 | 0.3419 | 1.2452 |
| ZINC02130274 | -10.6 | 7.77 | 0.2789 | 1.2452 |
| ZINC02133359 | -10.6 | 7.77 | 0.2789 | 1.2452 |
| ZINC02159223 | -10.6 | 7.77 | 0.3312 | 1.2452 |
| ZINC06137732 | -10.6 | 7.77 | 0.2789 | 1.2452 |
| ZINC06233831 | -10.6 | 7.77 | 0.3312 | 1.2452 |
| ZINC03839360 | -10.6 | 7.77 | 0.2944 | 1.5565 |
| ZINC03839422 | -10.6 | 7.77 | 0.2944 | 1.5565 |
| ZINC03849281 | -10.6 | 7.77 | 0.3118 | 1.5565 |
| ZINC01737612 | -10.6 | 7.77 | 0.3118 | 1.8678 |
| ZINC02127993 | -10.6 | 7.77 | 0.2865 | 1.8678 |
| ZINC04045796 | -10.5 | 7.7 | 0.3281 | 0.3113 |
| ZINC04236005 | -10.5 | 7.7 | 0.3 | 0.6226 |
| ZINC03839432 | -10.5 | 7.7 | 0.2838 | 0.9339 |
| ZINC03839450 | -10.5 | 7.7 | 0.3 | 0.9339 |
| ZINC03882093 | -10.5 | 7.7 | 0.3621 | 0.9339 |
| ZINC04237101 | -10.5 | 7.7 | 0.3182 | 0.9339 |
| ZINC02117992 | -10.5 | 7.7 | 0.3182 | 1.2452 |
| ZINC02119947 | -10.5 | 7.7 | 0.3281 | 1.2452 |
| ZINC02130599 | -10.5 | 7.7 | 0.3 | 1.2452 |
| ZINC02130647 | -10.5 | 7.7 | 0.3182 | 1.2452 |
| ZINC02132169 | -10.5 | 7.7 | 0.2838 | 1.2452 |
| ZINC02135464 | -10.5 | 7.7 | 0.3281 | 1.2452 |
| ZINC02148976 | -10.5 | 7.7 | 0.3182 | 1.2452 |
| ZINC04222163 | -10.5 | 7.7 | 0.2838 | 1.2452 |
| ZINC03839423 | -10.5 | 7.7 | 0.2763 | 1.5565 |
| ZINC03849288 | -10.5 | 7.7 | 0.3088 | 1.5565 |
| ZINC04259704 | -10.5 | 7.7 | 0.2917 | 1.5565 |
| ZINC02096111 | -10.5 | 7.7 | 0.2838 | 1.8678 |
| ZINC04236176 | -10.5 | 7.7 | 0.2838 | 2.1791 |
| ZINC02106926 | -10.4 | 7.63 | 0.3059 | 0.3113 |
| ZINC00488832 | -10.4 | 7.63 | 0.4 | 0.6226 |
| ZINC00839387 | -10.4 | 7.63 | 0.3586 | 0.6226 |
| ZINC04236041 | -10.4 | 7.63 | 0.2971 | 0.6226 |
| ZINC05413670 | -10.4 | 7.63 | 0.325 | 0.6226 |
| ZINC03839373 | -10.4 | 7.63 | 0.2971 | 0.9339 |
| ZINC04236030 | -10.4 | 7.63 | 0.2971 | 0.9339 |
| ZINC04236421 | -10.4 | 7.63 | 0.2737 | 0.9339 |
| ZINC02113936 | -10.4 | 7.63 | 0.2811 | 1.2452 |
| ZINC02114512 | -10.4 | 7.63 | 0.3355 | 1.2452 |
| ZINC02120710 | -10.4 | 7.63 | 0.3355 | 1.2452 |
| ZINC02123774 | -10.4 | 7.63 | 0.325 | 1.2452 |
| ZINC02128142 | -10.4 | 7.63 | 0.325 | 1.2452 |
| ZINC02128671 | -10.4 | 7.63 | 0.325 | 1.2452 |
| ZINC02130273 | -10.4 | 7.63 | 0.2737 | 1.2452 |
| ZINC02133691 | -10.4 | 7.63 | 0.3059 | 1.2452 |
| ZINC02134714 | -10.4 | 7.63 | 0.325 | 1.2452 |
| ZINC02137525 | -10.4 | 7.63 | 0.3355 | 1.2452 |
| ZINC04222214 | -10.4 | 7.63 | 0.2737 | 1.2452 |
| ZINC04235929 | -10.4 | 7.63 | 0.3152 | 1.2452 |
| ZINC04235987 | -10.4 | 7.63 | 0.2889 | 1.2452 |
| ZINC05441286 | -10.4 | 7.63 | 0.2811 | 1.2452 |
| ZINC01105783 | -10.4 | 7.63 | 0.2889 | 1.5565 |
| ZINC02091598 | -10.4 | 7.63 | 0.2971 | 1.5565 |
| ZINC02120627 | -10.4 | 7.63 | 0.2889 | 1.5565 |
| ZINC02133402 | -10.4 | 7.63 | 0.3152 | 1.5565 |
| ZINC02138166 | -10.4 | 7.63 | 0.3152 | 1.5565 |
| ZINC02160994 | -10.4 | 7.63 | 0.3152 | 1.5565 |
| ZINC02119752 | -10.4 | 7.63 | 0.2971 | 1.8678 |
| ZINC02127995 | -10.4 | 7.63 | 0.2811 | 1.8678 |
| ZINC01431325 | -10.3 | 7.55 | 0.3433 | 0.6226 |
| ZINC04064925 | -10.3 | 7.55 | 0.3121 | 0.6226 |
| ZINC04237421 | -10.3 | 7.55 | 0.2943 | 0.6226 |
| ZINC05396219 | -10.3 | 7.55 | 0.3029 | 0.6226 |
| ZINC05396456 | -10.3 | 7.55 | 0.3029 | 0.6226 |

**Top hits of 100 compounds obtained out of 22000 compounds.*
