# Supplementary material for: Structure-guided identification of mitogen-activated protein kinase-1 inhibitors towards anticancer therapeutics
Source: PLoS One. 2025 Jan 24;20(1):e0311954. doi: 10.1371/journal.pone.0311954 (PMC11760640; doi:10.1371/journal.pone.0311954)
Supplement: S2 Table — (DOCX) [file pone.0311954.s002.docx]

**Table S2**: Selected hits and a control molecule with their structures.

| **S. No.** | **Ligand** | **2D structure** |
| --- | --- | --- |
|  | ZINC02161110 | 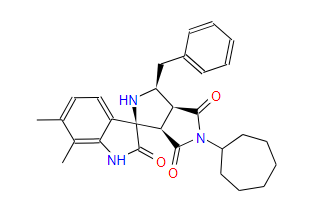 |
|  | ZINC02161108 | 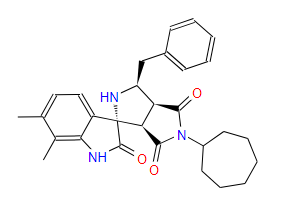 |
|  | ZINC03844856 | 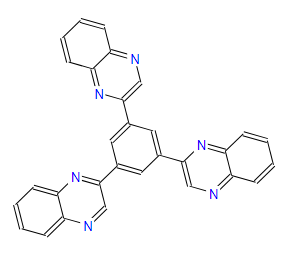 |
|  | ZINC02092851 | 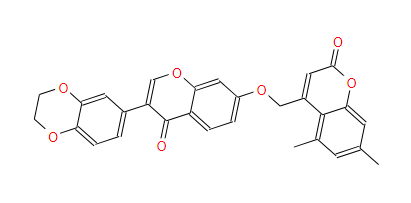 |
|  | ZINC02161106 | 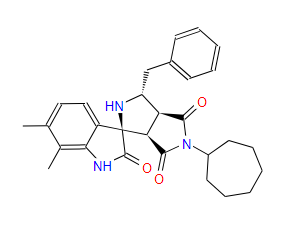 |
|  | ZINC02119958 | 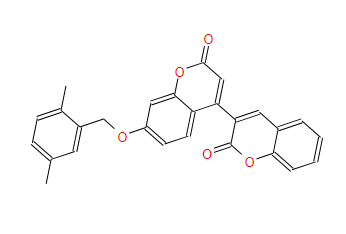 |
|  | ZINC04083885 | 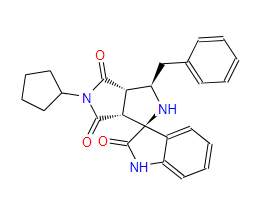 |
|  | ZINC03839446 | 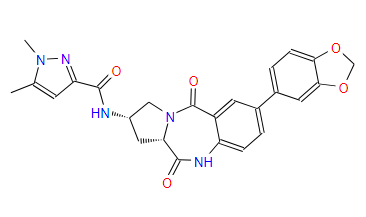 |
|  | ZINC02130647 | 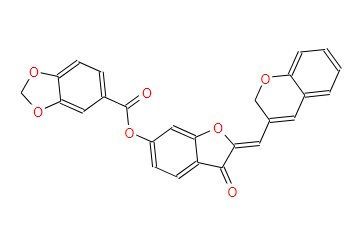 |
|  | ZINC02133691 | 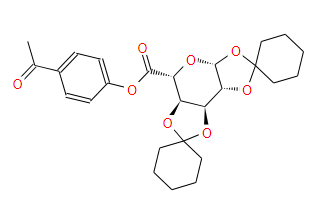 |
|  | Ulixertinib (control) | 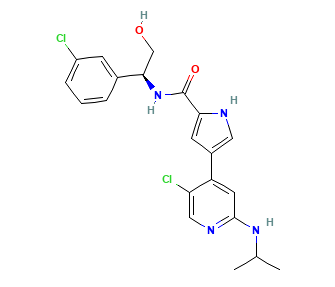 |
