# Supplementary material for: Structure-guided identification of mitogen-activated protein kinase-1 inhibitors towards anticancer therapeutics
Source: PLoS One. 2025 Jan 24;20(1):e0311954. doi: 10.1371/journal.pone.0311954 (PMC11760640; doi:10.1371/journal.pone.0311954)
Supplement: Attachment — Submitted filename: Response_Letter.docx [file pone.0311954.s003.docx]

**Journal:** PLoS One

**Manuscript Title:** Structure-guided identification of mitogen-activated protein kinase-1 inhibitors towards anticancer therapeutics

**Manuscript ID:** PONE-D-24-32925

**Reviewer #1**

In the submitted manuscript, the authors perform a major high throughput virtual screening of most of the major small molecule database (ZINC database) for Mitogen-activated protein kinase 1 (MAPK1), followed by further refinement of the results of structure-based virtual screening to molecular docking, and molecular dynamic simulation studies for up to 200ns. The manuscript is well written and easy to get through though with appropriately cited references. However, the major revision is required to reach the desired conclusions. The following are the major shortcomings in the manuscript that will require further work done -

1. Authors have chosen the MAPK1 structure (PDB ID: 8AOJ) for this study. The authors should justify their use of the MAPK1 for screening of inhibitors for therapeutic use in a human disease.

***Response:*** *Thank you for your suggestion. Our revised manuscript now justifies the use of MAPK1 for screening inhibitors.*

2. Does author refined the structure obtained from the PDB ID: 8AOJ? The authors should justify this in the manuscript by highlighting structural features of MAPK1 and adding refinement part to the manuscript.

***Response:*** *Thank you for your comments. Yes, the structure obtained from PDB was then refined by using all the coordinates and examining the structure and heteroatoms. Water molecules and co-crystallized ligands were removed from the initial coordinates. And the structure was remodelled to remove the missing residues by using PyMOD3.*

3. Despite having quite different structures in comparison to other MAPK, have very similar binding pockets and their ligands. In this case, how did the authors ensure that the MAPK1 ligands identified by them are specific only for this receptor and do not bind the other MAPKs? The authors should claim pharmacological significance of their findings in terms of specificity of their identified ligands among the MAPK.

***Response:*** *Thank you for your valuable feedback. We appreciate your suggestion. We have conducted extensive analysis that demonstrates the selective affinity of our ligands for MAPK1. The results indicate minimal off-target interactions with other kinases. these ligands modulate downstream signaling pathways, leading to distinct cellular responses, including altered proliferation, differentiation, and apoptosis rates. Compared with an existing MAPK1 inhibitor such as Ulixertinib, our ligands exhibit superior specificity and efficacy, with a favorable safety profile, reducing potential side effects. specificity of our ligands is due to unique binding interactions and conformational changes within the MAPK1, which we have detailed in interaction analysis section.*

4. Clearly justify the novelty in at least top 10 ligands listed in the Table 2. Why they were chosen? During their structure-based screening protocol the authors finally prioritize the ligands based on their interaction with MAPK1. However, there is no clear rationale given on the selection of these compounds.

***Response:*** *Thank you for your comments. Table 2 has now been justified in the updated manuscript.*

5. Also do the authors seek to claim that a known MAPK1 inhibitor identified until now bind to same site. What special advantage does a hydrogen bond to the catalytically important residues provide to ligand binding that cannot be matched by any other alternative hydrogen bonding in the binding site? The authors should justify this choice through further computational analysis or by citing references from literature that claim the importance of H-bonding network in the light of binding affinity and selectivity. Authors should discuss the same in their results.

***Response:*** *Thank you for your query. Hydrogen bonds to catalytically important residues can stabilize the transition state of the enzymatic reaction, thereby lowering the activation energy required for the reaction to proceed. This stabilization is crucial for enhancing the efficiency of the interaction. These hydrogen bonds often result in stronger interactions between the ligands and the target protein, leading to higher binding affinity. This is because the catalytically important residues are typically located in key positions within the active site, where they can form optimal interactions with the ligand. Also, they can contribute to the selectivity of the ligand for the target protein. By interacting specifically with these critical residues, the ligand is less likely to bind to other non-target proteins, reducing off-target effects. These hydrogen bonds can help maintain the proper conformation of the enzyme’s active site, ensuring that it remains in an optimal state for the interaction. This conformational stability is essential for the protein’s function and can be disrupted by alternative hydrogen bonds that do not involve the catalytically important residues.*

6. The authors should perform a ligand-receptor hydrogen bond occupancy analysis and perform distance calculations for the same, to show the percent occupancy of all hydrogen bonds claimed in 200 ns and plot their distances trajectory for the 200ns simulation with the donor/acceptor atoms on the receptor.

***Response:*** *We appreciate your suggestion. The ligand-receptor hydrogen bond analysis is performed in Figure 7 and discussed in the manuscript.*

7. Authors should provide the free energy interpretations of the Principal Component Analysis performed by them (Figure 9).

***Response:*** *Thank you for your feedback. The principal component analysis in terms of Gibbs free energy is well interpreted in the free energy landscape. In a free energy landscape, the color gradients represent the Gibbs free energy (G) in kilojoules per mol (kJ/mol). Blue/Green regions are the areas of low Gibbs free energy, corresponding to stable states or conformations that the system is most likely to occupy. These are often interpreted as local or global minima in the free energy landscape. Red regions represent the area of high Gibbs free energy, indicating less favourable or unstable states that the system is less likely to occupy.*

8. Technically well done including MD simulation time. However, what is missing is more discussion of the findings. Since the authors perform a 200ns simulation, it will be good if they provide deeper insights into the binding modes, receptor occupancy and mode of inhibition of the MAPK1 by the novel ligands they claim to identify.

***Response:*** *Thank you for your comment. Now, we have covered these key points in our revised manuscript.*

9. Authors should ensure that the references point out to the original publications associated with the tools and parameters and other unnecessary wordings and citations should be avoided to direct the reader clearly to the original methodology and parameter selection citations.

***Response:*** *All the citations have been thoroughly checked, and the unnecessary wordings are corrected now.*

10. Authors should go through the complete methodology section and double check all citations to ensure that every methodology citation goes back to the original published method or its updated version that was actually used in practice.

***Response:*** *Thank you for your suggestion. All the citations have now been thoroughly checked and verified.*

**Reviewer #2**

The manuscript titled “Structure-guided identification of mitogen-activated protein kinase-1 inhibitors” to identify novel therapeutic candidates against MAPK1. In my opinion, the manuscript needs to be improved with proper analyses and better representations. Several comments/suggestions for authors:

1. The introduction lacks several additions. Please describe in detail why you are trying to block the MAPK1 activity by explaining how it is implicated in each disease form and the side effects of each inhibitor.

***Response:*** *Thank you for your suggestion. The introduction part has been updated now.*

2. Highlight the reasons why authors chose protein structure with PDB code 8AOJ.

***Response:*** *The structure (PDB ID: 8AOJ) was chosen based on its higher resolution (1.12 Å), wild type in nature, and the query coverage of the desired kinase domain of MAPK1. The 8AOJ structure contains the kinase domain of the MAPK1 protein, and it has the highest resolution among the X-ray crystallography structures.*

3. Which tools were used to calculate the number of hydrogen bonds? Provides more information for the bonding angle between the hydrogen donor (D) and acceptor (A) D-H⋯A and the distance between D and A.

***Response:*** *PyMOL and Discovery Studio Visualizer were used to calculate hydrogen bonds. The angle and distance cut-off for hydrogen bonds between donor (D) and acceptor (A) were set to 3.5 Å and 150-180°, respectively. Now, we have included the bond angle and distance in the revised manuscript.*

4. The authors are advised to provide the hydrogen bond occupancy.

***Response:*** *Hydrogen bond occupancy analysis has been included in the updated manuscript.*

5. The number of hydrogen bonds is very high in the MD simulation plot, however, it is quite low in docking. It needs to be analyzed properly.

***Response:*** *The number of hydrogen bonds in MD simulation plots are intramolecular hydrogen bonds within MAPK1. The intermolecular hydrogen bonds between compounds and the protein are plotted and discussed in Figure 7 of the manuscript.*

6. The authors need to explain Table 3 in the manuscript text. Please provide optimal reference values for all attributes.

***Response:*** *Thank you for your feedback. Table 3 has been elaborated now.*

7. Why did the authors choose the SPC water model for simulations?

***Response:*** *Thank you for your query. We used the TIP3P water model for simulations and corrected this mistake in our revised manuscript.*

8. Please mention the number of Na/Cl ions used specifically in the MD Simulations section.

***Response:*** *Thank you for your comment. Four Na^+^ ions were used in the MD Simulations and we have now mentioned the same in the revised manuscript.*

9. The manuscript should be further improved by adding previously published reference articles on MAPK1 inhibition that reflect in silico studies demonstrating the same key residues that the authors have obtained compared with previous studies.

***Response:*** *Thank you for your concern. The revised manuscript includes the previously published reference articles on MAPK1 inhibition.*

10. Please check the identified hits in the PubChem registry to see if they have been evaluated before against MAPK1 and mention the same in the manuscript.

***Response:*** *The identified compounds have been verified. These compounds have not been evaluated against MAPK1. The revised manuscript has updated this information.*

11. Authors should include the 2D structures of both hit compounds along with their 2D structures for comparison and also report if the two structures have similar subgroups as part of their structural assessment.

***Response:*** *Thank you for your valuable suggestion. The 2D structures for both hits have now been included in Table 1. Yes, a few structures have similar subgroups, and they were eliminated accordingly.*

**Reviewer #3**

MAPK1 is a critical target for cancer therapy due to its central role in various cancers. In search of novel MAPK1 inhibitors, the authors conducted a comprehensive study involving virtual screening, molecular dynamics (MD) simulations, and essential dynamics analysis. Using the ZINC database, they screened a library of natural compounds and identified three promising candidates: ZINC02092851, ZINC02130647, and ZINC02133691. These compounds demonstrated efficient binding and favorable structural orientation within the MAPK1 binding pocket, showing strong affinity and specificity for active site residues. MD simulations revealed the stability and dynamic behavior of these compounds, both as complexes and in the free state of MAPK1, with no significant fluctuations observed. The identification of these compounds marks a significant advancement, suggesting their potential as MAPK1 inhibitors and emphasizing the importance of natural product-based drug discovery. During revision, following changes are necessary to enhance the quality of manuscript.

1. The abstract provides a clear overview, but it lacks a brief mention of the potential clinical implications of the findings. Including a sentence on the relevance of the identified compounds in the context of developing new anticancer drugs would enhance the abstract.

***Response:*** *The abstract part has now been updated.*

2. The introduction adequately highlights the significance of the current research and the need for novel drug targets. Consider expanding on the current status of drug development and how the study contributes to addressing this challenge.

***Response:*** *Now the introduction has been extended on the current status of drug development during this revision.*

3. Provide more details on the criteria used for selecting the compounds for analysis. This information is essential for the reproducibility of the study.

***Response:*** *The revised manuscript now includes the selection criteria for the identified compounds.*

4. Include a brief explanation of the molecular docking technique and parameters used, ensuring clarity for readers less familiar with computational approaches.

***Response:*** *Thank you for your comment. The molecular docking technique has now been explained properly.*

5. Elaborate on the molecular dynamics simulation protocol, specifying force field details, simulation parameters, and software tools employed.

***Response:*** *Thank you for your suggestion. Molecular dynamic simulation has been explained properly in our revised manuscript.*

6. Clearly present the results of the molecular docking analysis, including the binding energies of the top compounds and their potential affinities for MAPK1.

***Response:*** *Molecular docking analysis has been clearly demonstrated in the result section.*

7. Provide a comprehensive overview of the molecular dynamics simulations, emphasizing the stability of the identified compounds over the simulation period. Highlight any noteworthy interactions observed during the simulations.

***Response:*** *Thank you for your comments. In molecular dynamics simulations, RMSD, RMSF, Rg, SASA, dynamics of hydrogen bondings, and free energy landscape, e.t.c. are the key parameters to analyse the structural stability and compactness of the protein-ligand complexes. Our study found that the MAPK1-ZINC02130647 complex exhibited the lowest average RMSD value (0.24 nm) compared to the other complexes (MAPK1-ZINC02092851 at 0.31 nm and MAPK1-ZINC02133691 at 0.26 nm), indicating greater structural stability. Additionally, the MAPK1-ZINC02130647 complex showed lower RMSF values as well, suggesting reduced flexibility and enhanced stability upon binding with MAPK1 as compared to the other two complexes. Furthermore, this complex also demonstrated the lowest Rg value, reflecting a more compact structure, which often correlates with increased stability. Overall, MAPK1-ZINC02130647 emerged as the most stable among all the complexes, suggesting that ZINC02130647 forms a stronger and more stable interaction with MAPK1 compared to the other ligands, potentially making it a more promising candidate for inhibiting MAPK1 activity. We have discussed this in the revised manuscript.*

8. Interpret the significance of the findings in the context of existing literature on known MAPK1 inhibitors.

***Response:*** *Thank you for your comment. Our findings, ZINC02092851, ZINC02130647, and ZINC02133691 as MAPK1 inhibitors, can be more significant as they showed improved binding affinity, selectivity, novel mechanisms of action, better structural insights, enhanced efficacy in biological models, improved pharmacokinetics, and effectiveness against MAPK1 as compared to that of a known inhibitor,* *Ulixertinib.*

9. Discuss the limitations of the study, such as any assumptions made during the computational analysis, and propose avenues for future research.

***Response:*** *The potential limitations of the study have been discussed in the manuscript.*

10. Summarize the key findings and emphasize the relevance of the identified compounds as potential inhibitors of MAPK1 in the conclusion.

***Response:*** *Thank you for your suggestion. The conclusion has been updated now.*

11. Carefully proofread the entire manuscript to correct grammatical and typographical errors. Ensure consistent formatting and citation style throughout the manuscript.

***Response:*** *The revised manuscript was proofread before submission.*

12. Ensure that figures and tables are appropriately labeled and referenced in the text.

***Response:*** *We checked and ensured that all the figures and tables were appropriately labelled and referenced.*

**Reviewer #4**

In this submission, authors employed a structure-guided virtual screening of natural compounds from the ZINC database, initially filtered using the Lipinski rule of five to ensure drug-likeness. Molecular docking was then performed to evaluate the binding affinity and specificity towards the MAPK1 kinase domain. Three compounds, ZINC0209285, ZINC02130647, and ZINC02133691, emerged as promising candidates due to their significant binding potential and favorable drug-like properties. These compounds underwent rigorous all-atom molecular dynamics simulations over 200 nanoseconds to assess their stability and interactions within the MAPK1 kinase domain. Our findings suggest that these compounds hold potential as therapeutic candidates against MAPK1-associated cancers, highlighting their promise for further development in targeting cancer metastasis. Despite, the manuscript is well structured, updated and interesting. However, there are some changes to be made that are required before acceptance of the paper:

1. The author can improve the introduction section by adding latest references and highlighting rationale of the study and hypothesis.

***Response:*** *Thank you for your suggestions. The latest references, as well as the rationale and hypothesis, have been added to the revised manuscript.*

2. It would be helpful to know the criteria the authors used to select the best binding pose of the ligands. How can the authors be certain that the top binding pose among the possible poses is the correct one?

***Response:*** *Thank you for your suggestion. The direct way to analyse the best binding pose is the docking score provided by docking software. The docking score estimates the binding affinity of the ligand and the target protein based on the factors like van der Waal, hydrogen bonding, electrostatic interactions and hydrophobic effect. Another method for selecting the best pose is interaction analysis. In this, the selected pose should display key interactions with critical residues in the binding site, such as hydrogen bonds, hydrophobic contacts, etc, that are known to be essential for biological activity.*

3. The authors should provide a detailed discussion on the binding of the top three compounds, i.e., ZINC0209285, ZINC02130647, and ZINC02133691, showed a remarkable drug likeliness with promising binding potential towards MAPK1.

***Response:*** *Thank you for your feedback. The selected compounds, ZINC02092851, ZINC02130647, and ZINC02133691, demonstrated high affinity and specificity for the ATP-binding pocket of MAPK1 and drug-likeness properties. The MD simulation results indicated that the binding of both compounds stabilizes the MAPK1 structure and leads to fewer conformational changes. Our findings suggest that ZINC02092851, ZINC02130647, and ZINC02133691 may be potential inhibitors for MAPK1, offering valuable leads for drug development against MAPK1-associated cancers.*

4. The authors should describe the MD results in the context of best compounds considering that ZINC0209285, ZINC02130647, and ZINC02133691 have not moved from their initial docking positions on CDK8. This can be explained by evaluating the intermolecular hydrogen bonding between the compounds with MAPK1.

***Response:*** *Intermolecular hydrogen bonding between the selected compounds and MAPK1 has been performed and the interactions are explained in our manuscript. Thanks!*

5. The average Rg values of the complexes in Figure 5 are consistently altered for those of the free protein. The authors should provide an explanation for this observation.

***Response:*** *Thank you for your query.* *The time-dependent assessment of Rg revealed that, throughout the simulation, MAPK1 maintained stability within the range of 1.8 nm to 1.9 nm in the presence of ZINC02092851, ZINC02130647, and ZINC02133691. Comparative analysis indicated that the folding of MAPK1 does not show any major fluctuation after binding with ZINC02092851, ZINC02130647, and ZINC02133691. Further supporting this observation, the PDF plot demonstrated similar distributions of Rg values for MAPK1 before and after.*

6. In order to facilitate a clearer comparison of the RMSF data, it would be beneficial if the authors could estimate the average RMSF values and include them in their analysis.

***Response:*** *Thank you for your comment. The manuscript mentions the average RMSF values of free MAPK1 and its complexes.*

**7.** The manuscript should elaborate on the relationship between the conformational changes observed in the enzyme and its inhibitory function, as mentioned in the study.

***Response:*** *Thank you for your valuable suggestion. The interaction analysis section of our revised manuscript elaborates on conformational changes in enzymes and their inhibitory functions.*

8. It would be beneficial for the authors to discuss the significance of findings in relation to protein-ligand binding.

***Response:*** *Thank you for your suggestion. The significance of the findings has been added to the revised manuscript.*

9. The authors should discuss the limitations of the study (in-vitro or in-vivo experimentation), such as the accuracy of the virtual screening and molecular dynamic simulation methods used, and the potential challenges of translating the findings to clinical settings.

***Response:*** *Thank you for your comment. An additional section for the limitations of computational studies has been included in the revised manuscript.*

10. The section conclusion should be modified to give a clear understanding of the paper concisely.

***Response:*** *Thank you for your suggestion. Conclusion has been modified now.*

11.  Many abbreviations are not described at first appearance. Author should recheck.

***Response:*** *Thank you for your comment. All the abbreviations have now been thoroughly checked.*

12. All references should be thoroughly checked, and especially Author must confirm only relevant publications should be cited.

***Response:*** *All the references have been verified now.*

13. The manuscript could benefit from additional proofreading and editing to ensure clarity and precision in the presentation of the results and conclusions.

***Response:*** *Thank you for your valuable suggestion. Proofreading has been done, and the manuscript has now been properly revised.*

**Reviewer #5:**

In the present manuscript authors have identified novel candidates against mitogen-activated protein kinase-1 though structure based virtual screening of 22000 compounds. The work is extensive and suitable for publication. However following queries needs to be resolved in revised manuscript.

1. The screening of compounds based on the docking score should be in comparison with standard. Here authors have not taken any standard. Docking studies of standard must be performed and compounds should screen based on comparison with standard.

***Response:*** *Thank you for your suggestion. Docking with a standard molecule has been carried out now, and the result has been included in Table 1 and the comparative discussion as well.*

2. Author should include limitations of computational studies for that following article may helpful and should be included with proper citation. https://doi.org/10.1016/B978-0-323-90608-1.00006-X, https://doi.org/10.1016/j.drudis.2020.07.005

***Response:*** *Thank you for your concern. The revised manuscript now includes limitations of computational studies with the respective citations.*

3. The ADMET analysis needs to be revise with proper depth analysis for that following article may helpful and should be included with proper citation. https://doi.org/10.1007/s42250-022-00376-7, https://doi.org/10.33263/BRIAC122.13851396

***Response:*** *Thank you for your valuable suggestion. ADMET analysis has been revised now.*

4. The discussion of previous studies of natural products explored against some important targets of cancer using insilico approach will provide more valuable insights. following article may helpful and should be included with proper citation.

https://doi.org/10.1007/s13205-023-03912-5, https://doi.org/10.3389%2Ffphar.2023.1236173,
https://doi.org/10.1007/s42250-023-00611-9, https://doi.org/10.1021%2Facsomega.2c04837
https://doi.org/10.2174/1573409916666200408082858
https://doi.org/10.1016/j.cbi.2024.110927

***Response:*** *The revised manuscript now includes a discussion of previous studies of natural compounds considering the suggested references.*
